# Private Genomes and Public SNPs: Homomorphic Encryption of Genotypes and Phenotypes for Shared Quantitative Genetics

**DOI:** 10.1534/genetics.120.303153

**Published:** 2020-04-22

**Authors:** Richard Mott, Christian Fischer, Pjotr Prins, Robert William Davies

**Affiliations:** * Genetics Institute, University College London, WC1E 6BT, UK; † Department of Genetics, Genomics and Informatics, University of Tennessee Health Science Center, Memphis, Tennessee 38103; ‡ Department of Statistics, University of Oxford, OX1 3LB, UK

**Keywords:** quantitative genetics, homomorphic encryption, genetic privacy

## Abstract

Sharing human genotype and phenotype data is essential to discover otherwise inaccessible genetic associations, but is a challenge because of privacy concerns. Here, we present a method of homomorphic encryption that obscures individuals’ genotypes and phenotypes, and is suited to quantitative genetic association analysis. Encrypted ciphertext and unencrypted plaintext are analytically interchangeable. The encryption uses a high-dimensional random linear orthogonal transformation key that leaves the likelihood of quantitative trait data unchanged under a linear model with normally distributed errors. It also preserves linkage disequilibrium between genetic variants and associations between variants and phenotypes. It scrambles relationships between individuals: encrypted genotype dosages closely resemble Gaussian deviates, and can be replaced by quantiles from a Gaussian with negligible effects on accuracy. Likelihood-based inferences are unaffected by orthogonal encryption. These include linear mixed models to control for unequal relatedness between individuals, heritability estimation, and including covariates when testing association. Orthogonal transformations can be applied in a modular fashion for multiparty federated mega-analyses where the parties first agree to share a common set of genotype sites and covariates prior to encryption. Each then privately encrypts and shares their own ciphertext, and analyses all parties’ ciphertexts. In the absence of private variants, or knowledge of the key, we show that it is infeasible to decrypt ciphertext using existing brute-force or noise-reduction attacks. We present the method as a challenge to the community to determine its security.

## Abstract (precis)

Mott *et al.* show that association between a quantitative trait and genotype can be performed using data that has been transformed by first rotating it in a high-dimensional space. The resulting...

WITH the growth of clinical genome sequencing, the number of individual human genomes available for analysis is increasing dramatically. To make the most of this resource, we need to be able to share and analyze genetic and phenotypic data securely.

The conflicting demands of individual privacy *vs.* medical research have led to a spectrum of ways of sharing human genotype and phenotype data (Azencott 2018). In a small minority of studies, anonymized data [that is, where the names of individuals have been replaced by anonymous identifiers (IDs)] are freely available for users to download and analyze. More usually—as for the UK BioBank and the UK 10k project, and studies deposited in the National Center for Biotechnology Information Database of Genotypes and Phenotypes and the European Bioinformatics Institute European Genome-phenome Archive (EGA)—anonymized data are distributed only to researchers approved for access, whose institutions demonstrate that their computer systems are secure, and where they agree not to redistribute the data. The host data archive then prepares data sets, encrypted with keys that may be specific to each data request, for transfer over a public network. After downloading the encrypted files within the firewall of the researcher’s computer system, they are decrypted into plain text. The advantage of this approach is that the researcher then has complete access to the anonymized genotypes and phenotypes, with only the identities of the samples being redacted; there is then no technical limitation as to the genetic analysis that can be performed. However, this carries certain risks because a data breach cannot be ruled out, and even if the data are anonymized, comparing anonymous genotypes with those of genotyped relatives might still reveal genetic relationships (Hansson *et al.* 2016).

At the other extreme, data sets are not distributed, but researchers may negotiate access to analyze the data on the host’s computer system (as in the UK 100,000 genomes project), or the host may agree to perform an analysis on behalf of an external user. No direct access to the raw data is granted, but analyses are shared. In still other cases, only the summary statistics of genome-wide association studies (GWAS) are distributed, typically comprising the regression coefficients and *P*-values of the genetic variants tested for association with the phenotype, for a federated meta-analysis. Such analyses combine sets of summary statistics from different GWAS, where participating laboratories have collected phenotypes and genotypes for different sets of subjects imputed at the same SNPs, and wish to test association across all studies (Pasaniuc and Price 2017).

Another approach that is gaining traction is to encrypt genotypes and phenotypes in such a way that it is still possible to perform relevant computations on the data, possibly on a remote or cloud computer, without decrypting them, *i.e.*, one can “throw away the key.” Homomorphic encryption (HE) refers to cryptographic systems that allow computations to be performed on encrypted data (the ciphertext) without decrypting it, and which yield the same answers as when the analogous computations are performed on the original data (the plaintext). It is an active area of research in computer science because it could make cloud computing much more secure, for both genetic and other applications. With HE, it is possible to build systems that store and process encrypted data, such that the data always stay encrypted both in transit and at rest. Should a cloud service be compromised, any stolen ciphertext would be valueless.

We define HE for genotypes and phenotypes (HEGP) to mean a transformation of the data that preserves those structures necessary for analysis while obscuring the individuals’ identities, phenotypes, and genotypes. Only the encrypted data are moved and shared between systems. HEGP is attractive because it enables testing genetic association across multiple data sets, in a federated mega-analysis based on the genotypes instead of a less powerful meta-analysis based on the summary statistics.

In the clinical field, methods such as the random time shifting of anonymized patient records (Hripcsak *et al.* 2016) offer some protection while not being cryptographically secure. In the quantitative genetics field, a number of approaches to HEGP have been proposed. In Jagadeesh *et al.* (2017) Yao’s protocol is used to identify rare Mendelian-type mutations shared between affected individuals. In Cho *et al.* (2018), secure multiparty communication is used to perform GWAS using principal components to control for population structure. Bonte *et al.* (2018), Tkachenko *et al.* (2018) and Sim *et al.* (2019) describe cryptographically secure protocols for computing *P*-values for case-control studies using contingency table χ^2^ tests. All these methods are thought to be cryptographically secure, but they limit the types of computation and data exploration possible. In particular, they cannot control for population structure using a mixed linear model, which is the current gold standard for quantitative trait analysis. In addition, they tend to be slower than analyses of unencrypted data.

Here, we consider whether linear transformations of genotypes and phenotypes can be used as keys for HE. The first class of transformations we investigate are random orthogonal transformations. These leave invariant essential parts of the linear mixed-model framework for complex trait analysis, preserving genotype correlations between SNPs while obscuring those between individuals. They share the same likelihood functions as unencrypted data. Any standard mixed-model type of analysis (including estimating heritability) will produce the same output as with unencrypted data. We ask if an orthogonal key can be generated in a sufficiently random manner to make the data unrecognizable, and show that keys sampled from the Steifel manifold have this property; however, not all orthogonal matrices make suitable keys. Once encryption has taken place, we show that computations are essentially identical to those using unencrypted data. They also can be extended to perform federated mega-analyses in a natural way. Their major drawbacks are that they are unsuitable for logistic regression and that the method is not provably secure. In particular, individuals with private variants are not securely encrypted by orthogonal transformation. However, for variants present in multiple individuals we present arguments that suggest it would be very challenging to find the key and hence decrypt the data.

The second type of linear transformation we consider is based on the mixed-model transformation. We show that this is likely to be more secure than orthogonal transformation but is more limited in its applications.

## Materials and Methods

### Data sets

We tested our encryption scheme on 10,640 individuals from the CONVERGE study of major depressive disorder (Cai *et al.* 2017), and on the smaller mouse data set of 1329 individuals and 19,877 SNPs from (Nicod *et al.* 2016) for platelet counts on mouse chromosome 11, which are publicly available as described below in Supplemental Material, File S1. We use the mouse data for the majority of the analyses in this study so that users may replicate our analyses by downloading the data and code.

### Data availability

The data and software are available from University College London figshare at https://rdr.ucl.ac.uk/articles/Mouse_Platelet_Dataset/11907687. In addition, the GitHub repository https://github.com/encryption4genetics contains the software and libraries (under an AGPLv3 free software license) and mouse data used in the study, as well as ongoing developments of the system. The authors state that all data necessary for confirming the conclusions presented in the manuscript are represented fully within the manuscript. Supplemental material available at figshare: https://doi.org/10.25386/genetics.8251535.

## Results

### Conceptual overview of quantitative genetic encryption

**Figure 1:**
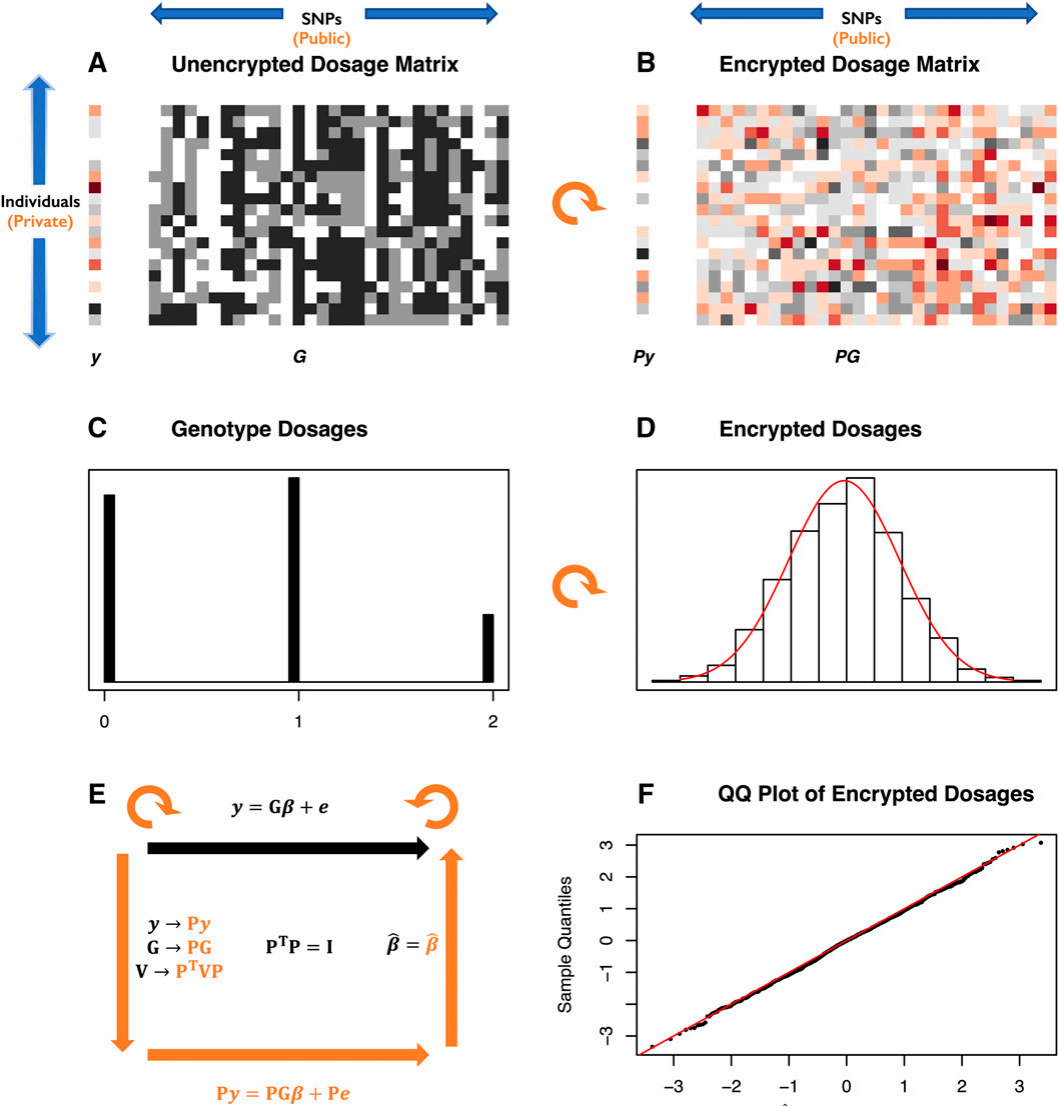
Privacy in relation to quantitative genetic analysis. (A) A numeric phenotype vector y (left) and genotype dosage matrix G (right) are represented as colors and shades of gray. Each row of the matrix represents one individual and each column one SNP. Genotypes are encoded as imputed dosages clustered at the values 0,1,2 giving the numbers of alternate alleles. (B) The same data after multiplication by an orthogonal matrix P (a rotation represented by the curved orange arrow). The genotype dosages are now represented by a continuum of real numbers. (C) The distribution of dosages for a particular SNP (column of G), clustered around 0,1,2. (D) The distribution of the same dosages after orthogonal transformation by multiplication by the orthogonal matrix P (black histogram) with the normal distribution with same mean and variance superimposed in red. (F) The normal QQ plot for the data in D, showing the transformed dosages are very close to a normal distribution. (E) A cartoon of the HEGP scheme. The top black arrow and equation show the linear mixed model relating the phenotype y to genotype G with regression coefficients β representing the allelic effects. The variance matrix for the residuals is V. After multiplication by orthogonal matrix P, data y, G and V and the mixed linear model are transformed as shown in orange. The likelihood and regression estimates $\hat{\beta }$ are preserved. HEGP, homomorphic encryption for genotypes and phenotypes; QQ, quantile–quantile.

The conflict between respecting individuals’ privacy and establishing allelic effects is sketched in Figure 1A. We have a vector of phenotypes, y, and a matrix of genotypes, G. Each row of the matrix corresponds to genotypes for a given individual, and each column to a given SNP. The phenotype and each genotype vector (column of G) are standardized to have mean 0 and variance 1. The standardized genotypes are dosages proportional to the estimated number of alternative alleles; a typical trimodal distribution of dosages is also shown in Figure 1C (for clarity they are shown prior to standardization). We want to preserve the privacy of the individuals (rows) but make public certain information about the effects of the SNPs (columns) in relation to each other and to the phenotype.

**Figure 2:**
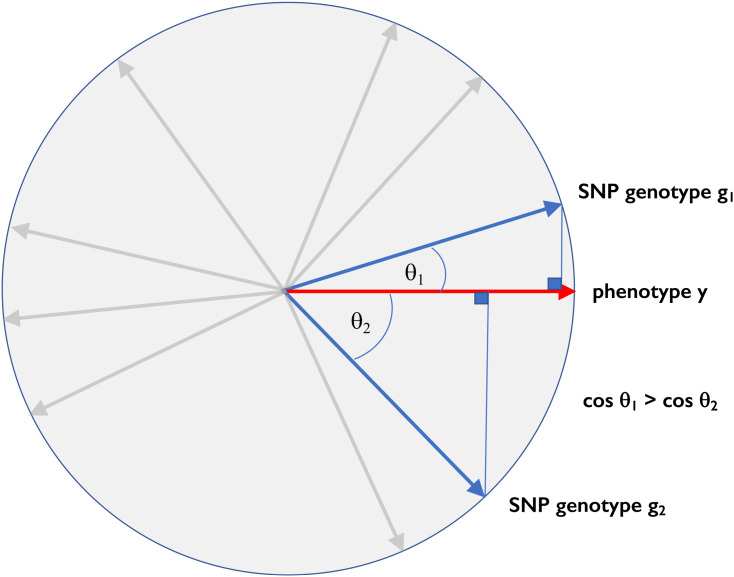
Geometric interpretation of genetic association. Phenotypes and genotypes are represented as unit vectors in a high-dimensional space. The cosines of the angles between the phenotype vector y and various SNPs equal the corresponding Pearson correlations, which are closely related to the *t*-statistics for testing association. In the example, SNP 1 has a smaller angle with the phenotype than SNP 2, and hence a stronger genetic association.

Conceptually, it is helpful to recall that the standardized genotype dosages for a given SNP across n subjects (a column in Figure 1A) can be thought of geometrically as a unit vector in n-dimensional space lying on the n-1 dimensional embedded unit hypersphere, and the standardized vector of phenotypes as another point on the same hypersphere (Figure 2). We measure the association between phenotype and SNP from the angle θ between their n-dimensional vectors. Their Pearson correlation coefficient (an invertible transformation of the t-statistic used to determine significance of a linear regression of phenotype on genotype dosage) is equal to their dot-product, *i.e.*, cosθ. Similarly, linkage disequilibrium $R^{2}$ between any pair of SNPs is the square of the cosine of the angle between the SNPs. It is intuitively obvious that any orthogonal transformation, a rotation or reflection of the space, will leave all the angles between unit vectors unchanged (Figure 2). Thus, all the associations between phenotype and genotypes, and correlations within genotypes, are preserved by orthogonal transformations. Figure 1B shows the phenotypes and genotypes after orthogonal transformation. Even though the original distribution of the genotypes dosages is trimodal (Figure 1C), the transformed genotypes resemble a sample from a normal distribution (Figure 1, D and F).

It follows that, if the encryption key is an n×n orthogonal matrix P of floating point values such that $PP^{T}=I$ (where $P^{T}$ is the transpose of P), then multiplication of the key with the genotype/phenotype matrix acts like a rotation (or reflection). In this way each SNP column is rotated by multiplication by the key and, as discussed below, if the key is sampled randomly, then the elements of each column vector of the resulting encrypted genotype/phenotype matrix are approximately normally distributed (Figure 1, D and F). We next show that these transformations preserve key components of the linear mixed model relating the phenotype to the genotypes (Figure 1E)

### Statistical preliminaries

#### Mixed-model quantitative genetic association:

To make this geometric intuition rigorous, we first review the core standard computations required for a mixed-model GWAS. Suppose we have n subjects and m SNPs, a quantitative phenotype vector y of length n, a n×p covariate matrix **X** (containing information about, *e.g.*, sex, age, environmental covariates, and principal components for controlling population structure), and an n×m genotype dosage matrix G in which the entries typically take the values 0,1,2, such that $G_{ij}$ is the number of alternate alleles for the genotype of subject i at SNP j (G can also represent imputed dosages without any change to the argument). It is necessary to standardize the genotype matrix into the matrix H such that

$$\begin{array}{c} H_{ij}=\frac{G_{ij}-2\pi_{j}}{\sqrt{2\pi_{j}\left(1-\pi_{j}\right)}} \end{array}$$ (1)

where $\pi_{j}$ is the minor allele frequency of the SNP j (alternatively, each vector of dosages can be standardized empirically by subtracting its sample mean and dividing by its empirical SD). The phenotype vector y and each column of X must also be standardized to have mean 0 and variance 1.

The n×n additive genetic relationship matrix (GRM)

$$K=\frac{1}{m}HH^{T}$$ (2)

is used to model the variance–covariance structure of the phenotype as

$$\text{Var}\left(y\right)=V=K\sigma_{g}^{2}+I\sigma_{e}^{2}$$ (3)

where $\sigma_{g}^{2},\sigma_{e}^{2}$ are the genetic and environmental variance components, and

$$h^{2}=\frac{\sigma_{g}^{2}}{\sigma_{g}^{2}+\sigma_{e}^{2}}$$ (4)

is the additive heritability. These variance components are typically estimated by restricted maximum likelihood (Yang *et al.* 2011). The linear model to test the significance of the SNP j is

$$y=X\alpha +h_{j}\beta_{j}+e$$ (5)

where α is a vector of fixed effects, $h_{j}$ is the jth column of H, $\beta_{j}$ is the regression coefficient for SNP j, and e is the residual, with variance matrix V.

The mixed model transformation

$$A^{-1}y=\left(A^{-1}X\right)\alpha +A^{-1}h_{j}\beta_{j}+A^{-1}e$$ (6)

converts the mixed model into an ordinary least squares problem in which the variance matrix is the identity, *i.e.*, $\text{Var}\left(A^{-1}e\right)=I$. Here, A is the matrix square root of V, *i.e.*, $A^{2}=V$, which can be computed efficiently by eigen decomposition of K, alongside the estimation of the variance components $\sigma_{g}^{2},\sigma_{e}^{2}$ (Kang *et al.* 2008).

The realized genetic relationship between individuals i,k is summarized as the matrix element $K_{ik}$ and the relationship (Pearson correlation coefficient) between SNPs j,l as the element $L_{jl}$ in the m×m matrix

$$L=\frac{1}{n}H^{T}H$$ (7)

#### Orthogonal transformations:

We wish to find an encoding of the genotypes, covariates, and phenotype such that their plaintexts are obscured, but such that we can compute all the above quantities and test association between genotypes and phenotypes using the same mixed model.

Consider the eigen decomposition of the variance matrix $V=E^{T}\Lambda E$ where E is an orthonormal matrix of eigenvectors and Λ the diagonal matrix of eigenvalues. These quantities are determined (up to permutation and rotation) by the matrix V. The (symmetric) matrix square root used in the mixed-model transformation is defined as

$$A=E^{T}\Lambda^{0.5}E$$ (8)

where $\Lambda^{0.5}$ is the diagonal matrix whose entries are the square roots of the eigenvalues.

Suppose P is *any* orthogonal n×n matrix, *i.e.*, so that $P^{-1}=P^{T}$. Then consider working with the transformed genotype matrix F=PH, phenotype vector z=Py, and covariate matrix W=PX in place of the plaintext. Such a transformation corresponds to finding a new coordinate system, so the rows (subjects) in the transformed space no longer correspond to individuals.

First note that

$$F^{T}F=H^{T}P^{T}PH=H^{T}H=nL$$ (9)

so the m×m SNP-relationship matrix L is preserved, while the GRM

$$FF^{T}=PHH^{T}P^{T}=mPKP^{T}$$ (10)

is transformed. In other words, linkage disequilibrium (as measured by Pearson correlation) between SNPs is unaltered, but since the original subjects are transformed, intersubject correlations are destroyed. In fact, since after orthogonal transformation each “subject” is a weighted combination of the originals, it is not meaningful to describe them as subjects. Nonetheless,

$$\text{Var}\left(z\right)=\text{Var}\left(\mathrm{Py}\right)=PVP^{T}=PKP^{T}\sigma_{g}^{2}+I\sigma_{e}^{2}$$ (11)

and hence the transformed phenotype has the same variance components $\sigma_{g}^{2},\sigma_{e}^{2}$ and heritability $h^{2}$, even though the GRM is transformed. Define B=AP. as the ciphertext analog of the plaintext mixed-model transformation. That is,

$$\text{Var}\left(B^{-1}z\right)=\text{Var}\left(P^{T}A^{-1}y\right)=P^{T}\text{Var}\left(A^{-1}y\right)P=P^{T}IP=I$$ (12)

and hence the ciphertext “rotated mixed model”

$$z=W\alpha +f_{j}\beta_{j}+Pe$$ (13)

that is expressed entirely using the ciphertext D(P)={z,W,F} is equivalent to the original plaintext model, and can be converted to ordinary least squares by multiplication by $B^{-1}$. Furthermore, the log-likelihood for the data (provided the errors are normally distributed) is invariant after orthogonal transformation. That is, using standard change-of-variable rules for **$y=P^{T}z$** for the multivariate normal distribution, and recalling that the determinant of an orthogonal matrix |P|=±1, then the plaintext log-likelihood for y:

$$-2\log l\left(\alpha ,\beta_{j},\sigma_{g}^{2},\sigma_{e}^{2}\right)={\left(y-\mathrm{X\alpha}-h_{j}\beta_{j}\right)}^{T}V^{-1}\left(y-\mathrm{X\alpha}-h_{j}\beta_{j}\right)+\log\left|V\right|+n\log\left(2\pi \right)$$ (14)

is identical to the log-likelihood for ciphertext z when evaluated at the same parameters:

$${\left(z-\mathrm{W\alpha}-f_{j}\beta_{j}\right)}^{T}{\left(\mathrm{PV}P^{T}\right)}^{-1}\left(z-\mathrm{W\alpha}-f_{j}\beta_{j}\right)+\log\left|P^{T}\mathrm{VP}\right|+n\log\left(2\pi \right)$$ (15)

Hence, all inferences about the parameters based on the likelihood are unaffected by the transformation. In particular, they yield identical maximum likelihood parameter estimates and *P*-values for likelihood-based tests of significance. Furthermore, any analyses based on linkage disequilibrium between SNPs are unaffected by the transformation. It is also possible to compute GRMs corresponding to subsets of SNPs (*e.g.*, per chromosome) from the transformed genotypes.

### Generalizations

Here, we sketch various generalizations to the orthogonal encryption scheme.

First, analyses that are unaffected by orthogonal transformation include the estimation of parameters by ridge regression, Least Absolute Shrinkage and Selection Operator (LASSO), or by **Henderson’s mixed model equations**. The proof for ridge regression follows from the observation that the ridge estimator

$${\hat{\beta }}_{ridge}={\left(X^{T}X+Ik\right)}^{-1}X^{T}y={\left({\left(\mathrm{PX}\right)}^{T}\left(\mathrm{PX}\right)+Ik\right)}^{-1}{\left(\mathrm{PX}\right)}^{T}\left(\mathrm{Py}\right)$$ (16)

for any orthogonal matrix P and ridge scale parameter k. The proof for Henderson’s equations follows in a similar way, as under orthogonal transformation any data matrix transforms as X→PX and any variance matrix as **$V\to \mathrm{PV}P^{T}$** (since Henderson’s model is a special case of a mixed model it also follows from Equation 6). Consequently, genomic prediction from estimated fixed effects (best linear unbiased estimator) and predicted random effects (best linear unbiased prediction) is also unaffected, provided of course we have access to some unencrypted genotypes with which to make predictions.

Second, dominance effects might be incorporated in the following way. The additive genotype dosage matrix G can be augmented in the usual way by a matrix T defined as

$$T_{ij}=\{\begin{array}{c} 0\text{if}G_{ij}=0\\ \;1\text{otherwise} \end{array}$$ (17)

representing a dominance effect. Then, any combination of additive and dominance effects can be modeled as a linear combination of G,T, so that Equation 5 that models the effect of SNP j becomes

$$y=X\alpha +h_{j}\beta_{j}+t_{j}\gamma_{j}+e$$ (18)

where **$t_{j}$** is the jth column of T and $\gamma_{j}$ is the dominance effect.

Multiplying by the orthogonal matrix P produces

$$Py=\left(\mathrm{PX}\right)\alpha +({\mathrm{Ph}}_{j})\beta_{j}+\left(Pt_{j}\right)\gamma_{j}+Pe\;$$ (19)

The rest of the development is similar to the purely additive case. Investigators would need to share both the transformed additive and dominance matrices. It is not clear if this would make decryption easier.

Finally, the major principal components of the genotype dosage matrix are sometimes included as covariates, in place of or in addition to fitting a mixed model, to further control for population structure and admixture. The n×m dosage matrix H has singular value decomposition $H=\mathrm{U\Sigma}V^{T}$, where U is the n×n orthogonal matrix of principal components, Σ is n×n diagonal, and $V^{T}$ is n×m orthogonal. Thus $F=\mathrm{PH}=\mathrm{PU\Sigma}V^{T}$. This means the principal components U of H are transformed to PU so that if necessary, the principal components of F may be calculated and included in the linear mixed model without explicitly including them as plaintext covariates to be transformed.

### Orthogonal HE

We propose that, if the orthogonal key P is appropriately sampled at random and independently of the plaintext data D(I)={y,X,H}, then it homomorphically encrypts D(I)→D(P), sufficient to allow full mixed-model GWAS without revealing the plaintext.

The Pearson correlation between a standardized vector x and Px is

$$\rho_{P}\left(x\right)=\frac{x^{T}\mathrm{Px}}{n-1}$$ (20)

Thus, provided P is “far” from the identity matrix I then we expect $\rho_{P}\left(x\right)$ to be distributed like the correlation of two random vectors. We found that an effective way to do this is to sample orthogonal matrices from the Stiefel manifold (*i.e.*, the Haar measure over the orthogonal group) (Hoff 2009), a uniform sampling distribution for orthogonal matrices (Anderson *et al.* 2005).

To investigate this experimentally, we sampled a 1000×1000 matrix $P_{1000}$ using the R library “rstiefel.” This uses the following scheme to simulate an orthogonal n×n matrix: (i) simulate an n×n matrix M whose entries are all iid N(0,1), (ii) compute the eigen decomposition of the symmetric matrix $M^{T}M=Q^{T}\mathrm{SQ}$ where Q is n×n orthogonal and S is diagonal with positive entries, and (iii) return the orthogonal matrix $P=MQ^{T}S^{-0.5}Q$ where $S^{-0.5}$ is the diagonal matrix whose elements are the reciprocals of the square roots of the eigenvalues.

Now the eigen decomposition of an orthogonal matrix can be written as

$$P=C^{-1}\exp\left(i\Theta \right)C$$ (21)

where C is a (nonorthogonal) matrix of eigenvectors and Θ is a diagonal matrix of angles, so that the eigenvalues exp(iΘ) are pairs of conjugate complex numbers on the unit circle or ±1. Then, for λ real, define the set of orthogonal matrices $P\left(\lambda \right)=C^{-1}\exp\left(i\lambda \Theta \right)C$, which vary smoothly between P(λ=0)=I and P(λ=1)=P.

Studying this set as λ varies lets us explore the encryption properties of a particular “linear direction” in the space of orthogonal matrices, starting at the identity matrix and passing through P [incidentally, the set P(λ) forms a subgroup of the orthogonal matrices, such that P(λ)P(μ)=P(λ+μ), with inverse $P{\left(\lambda \right)}^{-1}=P\left(-\lambda \right)$; this subgroup is of course isomorphic to the real numbers under addition].

**Figure 3:**
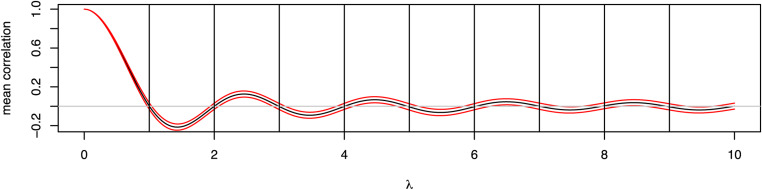
Correlation of unencrypted SNP dosages with encrypted versions as a function of λ. The black line shows the mean correlation ${\text{ρ}}_{\text{P}\left(\text{λ}\right)}\left(\text{x}\right)$ and the red lines the mean ± SD, estimated from 1000 individuals sampled from the CONVERGE study of major depressive disorder, at 1000 randomly chosen SNP sites.

Figure 3 shows the mean and SD of the correlation $\rho_{P\left(\lambda \right)}\left(x\right)$ for a 1000×1000 matrix $P_{1000}$ with 1000 SNPs sampled from the CONVERGE study of major depressive disorder (CONVERGE consortium 2015), for a subset of 1000 randomly sampled individuals. When λ=0, then the correlations are all unity, as would be expected, but as λ increases we observe a damped oscillatory behavior, with mean correlation of 0 at approximately λ=1,2,3,…. Thus, it is possible to sample a random orthogonal matrix such that, on average, there is no correlation between a random input vector of genotypes and its orthogonal transformation.

**Figure 4:**
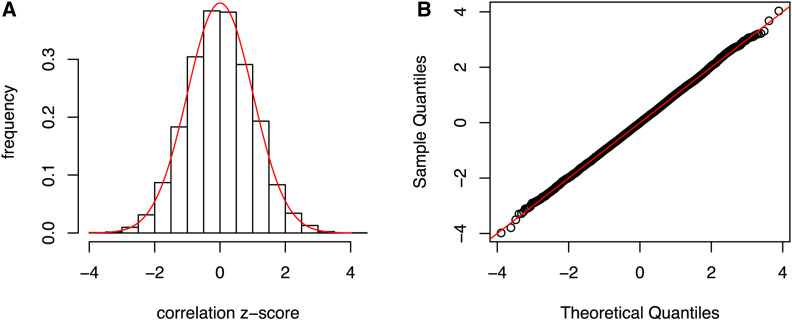
(A) The distribution of Z-transformed correlations ${\text{ρ}}_{10\text{k}}\left(\text{x}\right)$ evaluated at 10,000 randomly chosen CONVERGE SNPs. The red line is the normal density with the same mean and SD. (B) Normal quantile–quantile plot for the data in (A).

We applied these ideas to human genotype dosages from the CONVERGE study of major depressive disorder in n=10,465 individuals (CONVERGE consortium 2015). We generated a random 10,465×10,465 orthogonal matrix $P_{10k}$, which took ∼ 1 hr with two cores and 8 GB of memory. Figure 4A shows the distribution of the correlations $\rho_{10k}\left(x\right)$ evaluated at 10,000 randomly chosen SNPs, after Z-transformation $z=\rho \sqrt{\frac{n-2}{1-\rho^{2}}}$, and Figure 4B shows the QQ plot confirming the transformed correlations have the expected null normal distribution. Thus, transformed dosages are uncorrelated with their untransformed values, despite being a deterministic, invertible linear transformation of the latter. We return to this point later.

A potential concern is that rounding errors might arise due to the very large dimension of the key P. To test this, we computed $P^{T}P$, which should equal the identity matrix I. When $P=P_{10k}$, the off-diagonal values (which should all equal 0) had typical magnitude $10^{-11}$, indicating that the accuracy is acceptable. Nonetheless, the average magnitude of off-diagonal elements drifts upwards as the dimension of the matrix increases; when $P=P_{1000}$ the magnitudes are typically only $10^{-13}$. Therefore, we might eventually encounter rounding issues when sampling very large orthogonal matrices, but not for matrix dimensions up to at least 10,000. One solution would be to divide the samples into randomly chosen blocks of 10,000 individuals, sample a different transformation matrix to encrypt each block, and then permute all transformed data so that the block structure is hidden.

### Applications of orthogonal genotype encryption

It might be thought that orthogonal encryption is of little use, because both genotypes and phenotypes are transformed with the same orthogonal matrix, which must be known to those performing the transformation. However, there are uses for such a system. First, if the number of phenotypes is large (*e.g.*, from a gene expression study), then it might be necessary to analyze the data on an insecure cloud computing platform. Second, the encrypted data could be archived without special security concerns. Third, as we show next, it is possible to share and analyze federated independently transformed ciphertexts.

#### Sharing federated studies:

Suppose we wish to perform a federated mega-analysis on several genotype/phenotype sets. We assume that each set has first been imputed onto a common set of SNPs that are ordered consistently across data sets. Similarly, any covariates must be consistently defined and ordered across sets. Within each data set $D_{t}$, with $n_{t}$ subjects, an independent, private, orthogonal transformation is made using an $n_{t}\times n_{t}$ orthogonal matrix $P_{t}$ sampled at random to generate transformed ciphertext $D_{t}\left(P_{t}\right)$ as above. We combine the shared ciphertexts by stacking them top of each other. Thus, for three sets we have:

$$\begin{array}{c} D\left(I,z_{C},F_{C},W_{C}\right)=\left\{\begin{array}{c} D\left(z_{1},F_{1},W_{1}\right)\\ D\left(z_{2},F_{2},W_{2}\right)\\ D\left(z_{2},F_{3},W_{3}\right) \end{array}\right\}=\left\{\begin{array}{ccc} z_{1} & F_{1} & W_{1}\\ z_{2} & F_{2} & W_{2}\\ z_{2} & F_{3} & W_{3} \end{array}\right\}=\left\{\begin{array}{ccc} P_{1}y_{1} & P_{1}H_{1} & P_{1}X_{1}\\ P_{2}y_{2} & P_{2}H_{2} & P_{2}X_{2}\\ P_{3}y_{3} & P_{3}H_{3} & P_{3}X_{3} \end{array}\right\}\\ =\left(\begin{array}{ccc} P_{1} & 0 & 0\\ 0 & P_{2} & 0\\ 0 & 0 & P_{3} \end{array}\right)\left\{\begin{array}{ccc} y_{1} & H_{1} & X_{1}\\ y_{2} & H_{2} & X_{2}\\ y_{3} & H_{3} & X_{3} \end{array}\right\}=D\left(P_{C},y_{C},H_{C},X_{C}\right) \end{array}$$ (22)

where the subscript **C** denotes the combined data, and where the individual orthogonal matrices have been combined in a block-diagonal manner:

$$P_{C}=\left(\begin{array}{ccc} P_{1} & 0 & 0\\ 0 & P_{2} & 0\\ 0 & 0 & P_{3} \end{array}\right)$$ (23)

$P_{C}$ is orthogonal $\sum_{t}n_{t}\times \sum_{t}n_{t}$ and hence the combined data can be analyzed *as if* it were a single plaintext that had been encrypted using $P_{C}$. However, in reality, each laboratory contributing a data set $D_{t}$ independently encrypts their plaintext using their private key $P_{t}$ before sharing it.

Similarly, a data set could also be subdivided into subsets (*e.g.*, into male *vs.* female subjects) and each part encrypted separately so that subanalyses could be performed, and the subsets distributed separately. We emphasize that for federated analysis to work, it is necessary for the parties to agree in advance on a common set of SNPs and covariates.

#### Removing duplicates and close relatives: dual encryption:

One potential difficulty when sharing encrypted data is the possibility of duplicates or close relatives occurring in different cohorts. Because HEGP disguises genetic relationships, it would not be possible to identify duplicates in the shared ciphertexts. While there are simple practical ways of eliminating individuals with identical IDs in different studies (*e.g.*, first sharing the hashes of their sample IDs) or with identical genotypes at a small set of test SNPs (by sharing hashes of their genotype vectors), these methods would fail if the IDs were different or if the test genotypes differed even slightly (as might happen if samples were genotyped twice).

A solution would be for all parties to first agree on a restricted subset of $N_{R}$ common test SNPs (say 100 common SNPs chosen genome-wide). Each party computes a normalized plaintext $H_{R}$ restricted to just these SNPs, and they share the “dual ciphertext” $F_{R}=H_{R}P_{R}$, where **$P_{R}$** is a random $N_{R}\times N_{R}$ orthogonal key, instead of sharing **F=PH**.

Importantly, $F_{R}$ defines a dual form of encryption that has complementary properties to those of F; for the dual GRM

$$F_{R}F_{R}^{T}=H_{R}P_{R}P_{R}^{T}H_{R}^{T}=H_{R}H_{R}^{T}=N_{R}K_{R}$$ (24)

is the same as the plaintext GRM, while the SNP correlation matrix is scrambled. Dual encryption is therefore useless for genetic association. However, relatives and duplicates may be identified from the GRM $K_{R}$, and agreement reached on a revised subset of individuals from each study to be shared using the original scheme of encryption applied to all SNPs. However, it should be pointed out that sharing information in any way that reveals relationships between people is inherently risky.

### How secure is orthogonal encryption?

Can we determine P given only D(P)? Although we have shown that PH is uncorrelated with H, we have not shown that this renders the transformation truly secure. Since orthogonal encryption and decryption keys are essentially the same, our encryption has very different properties from public-key methods. Orthogonal encryption is certainly insecure for certain choices of P. As Figure 3 shows, any orthogonal matrix close to the identity matrix (*i.e.*, λ≈0) is clearly a poor choice, so one should restrict attention to random orthogonal matrices sampled from either the Stiefel manifold or using another scheme with similar sampling properties. One should also check that the mean of the correlations of the columns of F with the columns of H is close to zero.

It is obvious that any permutation of the rows of any key will transform the phenotype and genotypes in the same way, and so are functionally equivalent. Consequently, orthogonal permutation matrices in isolation are useless as keys. However, it also means that any permutation of any “good” key is also a good key.

The singular value decomposition of the unencrypted n×m dosage matrix H has $H=\mathrm{U\Sigma}V^{T}$, where U is n×n orthogonal, Σ is n×n diagonal, and $V^{T}$ is n×m orthogonal. Thus $F=\mathrm{PH}=\mathrm{PU\Sigma}V^{T}$, so the rotation U is simply replaced by another rotation PU. If P is truly random then we seek U given PU, which appears to be hard problem, since PU resembles another random orthogonal transformation.

Next, we discuss strategies that might be used to decrypt the data, in likely increasing order of effectiveness.

#### Brute-force:

We first tried sampling random decryption keys from the Stiefel manifold. Each key contains n(n+1)/2 independent double precision numbers, each of which can take ∼$10^{20}$ possible values. We defined a distance function between matrices as the mean of the absolute difference between each pair of corresponding elements (*i.e.*, the L1 norm), to compare the plaintext genotype matrix to an attempted decryption. We defined a good key as one that gives a mean distance of < 0.4 between the genotype matrix and the attempted decryption. Empirically this upper limit gave results that are visually fairly close to the original, at least for small data sets. Extrapolating from small matrices, we estimated a lower bound on the number of attempts required for solving an n×n key of one good key generated per $10^{n-1}$ incorrect keys. Thus, if n=100, ≥ $10^{99}$ keys have to be tried before a good one is found. Interestingly, even for an 8×8 matrix, we could not identify a key that regenerated the plaintext and even good keys did not reflect the underlying genotypes fully.

Generating orthogonal random keys is computationally expensive. The computational complexity of the Stiefel manifold is $O(n^{3})$; if n=100, a few hundred keys can be generated and evaluated per second on one CPU central processing unit (CPU) core. Our estimated bound suggests that it would take in the order of 10^92^ CPU hr to get close to a solution. Larger keys of realistic size take significantly longer, *e.g.*, when n=10,000, a single key takes ∼1 CPU hr to generate. Rather than generating orthogonal keys, a naive brute-force attack where potential keys are randomly selected would be even slower because the search space becomes much larger, including all nonorthogonal matrices. Thus, it takes a great deal of CPU power to guess large orthogonal matrices.

These experiments show that there is no consistent relationship between generated keys and their decryption outcomes using this simple metric. Moreover, the metric is defined in terms of distance to the plaintext, so it only works when we know the answer. In reality, an attacker would have to use a less-accurate score function. The number of possible permutations of the result matrix is so large that it is not feasible to use a brute-force attack without a method optimized to compute orthogonal matrices while optimizing for a metric that has an open-ended end result.

#### Exploiting non-Gaussian distributions of genotype dosages:

Another potential attack, that exploits specific features of the problem, is as follows. We note that the SNP identities (genomic positions) need to be distributed with the data to interpret the biology of any GWAS hits. Population allele frequencies for SNPs are generally available, and so for a SNP j with frequency $\pi_{j}$ that is in Hardy–Weinberg equilibrium, we expect to observe genotype dosages in the approximate proportions

$$0:\pi_{j}^{2},\;1:2\pi_{j}\left(1-\pi_{j}\right),2:{\left(1-\pi_{j}\right)}^{2}$$ (25)

After standardization, the dosages will be rescaled but will still be trimodal, with modes $d_{j0},d_{j1},d_{j2}$ that are completely determined by $\pi_{j}$ and the constraints that the standardized dosages have mean = 0, variance = 1 and that $d_{j0}-d_{j1}=d_{j1}-d_{j2}$.

Consequently, we might seek an orthogonal matrix approximation $\Phi \approx P^{-1}=P^{T}$ that maps F→ΦF with columns such that each has the frequency distribution close to that predicted by Hardy–Weinberg equilibrium. That is, for each SNP, the decrypted genotype dosages look like samples from a distribution with modes at $d_{j0},d_{j1},d_{j2}$ corresponding to the rescaled dosages 0,1,2 (like Figure 1C), which can be modeled using a kernel density estimate

$$\varphi_{j}\left(x,\tau \right)=\pi_{j}^{2}\phi \left(\frac{d_{j0}-x}{\tau }\right)+2\pi_{j}\left(1-\pi_{j}\right)\phi \left(\frac{d_{j1}-x}{\tau }\right)+{\left(1-\pi_{j}\right)}^{2}\phi \left(\frac{d_{j2}-x}{\tau }\right)$$ (26)

where ϕ(z) is a standard normal density kernel and τ is the SD of the kernel. Then, we seek an orthogonal matrix Φ∗ that maximizes

$$\begin{array}{c} \Phi *={\text{argmax}}_{\Phi }\prod_{j}\prod_{i}\varphi_{j}\left({\left(\mathrm{F\Phi}\right)}_{ij},\tau \right) \end{array}$$ (27)

We also require τ to be small to concentrate the data around the modes. However, if the plaintext dosages were imputed then they might well not be exactly integral, so it is necessary that τ>0 but is still as small as reasonably possible.

Equation 27 describes a nonconvex and nonlinear objective function. One potential approach to minimization is via robust nonconvex optimization based on the Cayley transform (Bertsimas *et al.* 2010; Wen and Yin 2013). Whether such an attack is feasible is unclear: the space of n×n orthogonal matrices has dimension n(n−1)/2, so if $n=10^{4}$, the minimization is over $4.995\times 10^{7}\approx 50$ million free parameters. There are likely to be local minima. It is also unclear if the minimizer Φ∗ is unique, or whether the true answer necessarily minimizes this quantity (by unique we mean if two distinct solutions Φ∗∗, Φ∗ exist then they are permutations of each other).

Fast Independent Components Analysis (FastICA) (Hyvärinen and Oja 1997) is another method that attempts to split non-Gaussian signals from Gaussian noise. FastICA (Hyvärinen and Oja 1997) finds an orthogonal transformation to map the data onto “interesting directions,” such that the projections of the data are strongly non-Gaussian along these directions; in our case, we seek directions in which the distributions are trimodal. In this context, FastICA may be thought of as maximizing a different function from the likelihood with a particular choice of optimization algorithm. However, we found that applying the implementation in the “fastICA” R package does not improve on our random brute-force attacks. We configured FastICA to produce an orthogonal matrix of the same size as the encryption key and computed the distance score of the resulting matrix. We found that these scores were much higher than the best keys generated during the brute-force attack. This is the case whether using a random initial matrix or providing an already generated key with a relatively good score. Table 1 shows the results of applying FastICA to the seven best keys from the brute-force attempt on a 4 × 4 key, with a 4 × 636 data set. The per-entry error in the decrypted data was 0.079 on average, which is quite good, while anything > 0.4 is unusable.

**Table 1:** Examples of attempted decryption using FastICA

Generated key score	Score of FastICA output initialized with generated key
0.07327044	0.39261006
0.07877358	0.44937107
0.08148585	0.39339623
0.08309748	2.67963836
0.08384434	0.4735456
0.08388365	2.67763365
0.08694969	2.82252358

Seven 4 × 636 genotype matrices were first encrypted and then attempts at decryption made either by brute-force random attempts (left column) or FastICA (right column). The score is the L1 distance between matrices. FastICA, Fast Independent Components Analysis.

As FastICA attempts to maximize non-Gaussianity in the attempted decryption, these results imply that non-Gaussianity does not describe the plaintext sufficiently uniquely. While the decrypted data are non-Gaussian, there are many other transformations of the ciphertext that also produce highly non-Gaussian results.

Another form of mathematical optimization is constrained convex programming, where constraints could be imposed to ensure the decrypted genotypes take plausible values. The main difficulty with applying convex programming (and linear programming, which also handles constraints) is the choice of a suitable objective function to minimize.

There is good reason to believe that nonconvex programming cannot produce good results. Optimizing a key to improve its decryption results would entail finding a path through the n-dimensional space of rotations, choosing both a correct direction to rotate in and a degree of rotation at each step. Specifically, the score function is not locally convex, and any naive optimization attempt is bound to fall into local minima. Similarly, gradient descent is also unlikely to be useful, as each iteration would require calculating a number (linear to the size of the key) of matrix multiplications (of the entire data set with the key at each step).

#### Compression:

The plaintext is highly compressible (at least if all the genotypes are integral), so we might instead seek

$$\begin{array}{c} \begin{array}{c} \Phi *={\text{argmin}}_{F}compress\left(\mathrm{F\Phi}\right) \end{array} \end{array}$$ (28)

where “compress” is some program like gzip. Again, we do not know if the most compressible encoding of the genotypes is identical to the true answer, or whether this could be computed efficiently. We expect that it would be very slow for large data sets.

#### Pedigrees:

If all the individuals in the study are from a set of known pedigrees (for example a large set of trios), then the expected plaintext GRM $E\left(K_{\mathrm{plaintext}}\right)$ is known (*i.e.*, entries for full siblings and parent–offspring will be one-half, those for unrelateds will be 0, etc.) and we can assume the samples are ordered so that the matrix is block diagonal. Then, the original and encrypted GRMs are linked via the approximation

$$E\left(K_{\mathrm{plaintext}}\right)\approx P^{T}K_{\mathrm{ciphertext}}P$$ (29)

which is a system of n(n+1)/2 quadratic equations in n(n+1)/2 independent unknowns (the number of degrees of freedom in an n×n orthogonal or symmetric matrix). Thus, an approximation to P could be obtained and might be a useful initial guess for further refinement, if n is small. When n is large, the problem has exponential time complexity (Grigoriev and Pasechnik 2005). Moreover, any permutation of the ordering of pedigrees that left $K_{\mathrm{plaintext}}$ unchanged would have the same solution so it would be impossible to assign phenotypes to pedigrees uniquely. Finally, if everyone is unrelated then $E\left(K_{\mathrm{plaintext}}\right)=I$ and the method would not work.

#### Incremental decryption:

Another way of thinking about the effects of the group of orthogonal transformations is that they define sets of equivalence classes of data sets D. That is, two sets $D_{1},D_{2}$ are equivalent if there exists an orthogonal matrix P such that $D_{1}\left(P\right)=D_{2}$, *i.e.*, that maps one to the other. The transitive property of the group of orthogonal matrices means that there is always an orthogonal matrix that will transform any pair of data sets provided they are in the same equivalence class. All data sets in the same equivalence class have the same likelihood, so these classes can be thought of as likelihood contours in a high-dimensional space.

This suggests another attack on the problem: find a series of N incremental orthogonal transformations that successively resolve individuals by “factorizing along the contour.” That is, we seek a sequence of orthogonal keys $\left\{\Phi_{k}\right\}$ and partially decrypted genotype matrices **$\left\{F_{k}\right\}$** such that (i) $\prod_{k}\Phi_{k}=P^{T}$ and (ii) $F_{k}\Phi_{k}\to F_{k+1}$ with **$F_{1}=F$**, **$F_{N}=H$**. There certainly exist infinitely many orthogonal keys that decrypt any subset of individuals. Suppose we want to decrypt the first k individuals. Then, if $Q_{n-k}$ is any n−k×n−k orthogonal matrix and $I_{k}$ is the k×k identity, and we partition $P=\left[P_{k}|P_{n-k}\right]$, such that $P_{k}$ is the **n×k** matrix comprising the first k columns of P, and $P_{n-k}$ is the last n−k columns, then

$$\left(\begin{array}{cc} I_{k} & 0\\ 0 & Q_{n-k} \end{array}\right)P^{T}=\left(\begin{array}{c} P_{k}^{T}\\ Q_{n-k}P_{n-k}^{T} \end{array}\right)=\left(\begin{array}{c} P_{k}^{T}\\ R_{n-k} \end{array}\right)$$ (30)

where $R_{n-k}$ is any **(n−k)×n** orthogonal matrix, will decrypt just the first k individuals. Thus, a sequence of matrices of the above form would decrypt the data. Using this scheme, in principle one could either try to decrypt individuals one-by-one in N=n steps or use a divide-and-conquer strategy with $N\approx \log_{2}n$ more difficult steps. Of course, since we do not know $P^{T}$ this merely proves existence: it is not clear that this type of approach is intrinsically better than trying to estimate $P^{T}$ directly; one still needs to estimate each column of $P^{T}$, a nontrivial task.

#### Private variants:

There is one clear-cut weakness to orthogonal encryption, which occurs when ultrarare private variants are present. Suppose a SNP j is private to the individual i. Then the genotype dosages for this SNP (column j of G) comprises n−1 zeros and one nonzero value, say 1 at row i. After standardization this pattern is preserved in H, although the numerical values are now scaled so the j’th column mean is zero and its variance is unity. The column j of PH is then

$$\begin{array}{c} {\left(\mathrm{PH}\right)}_{j}=\frac{\left(n-1\right)P_{i}-p}{\sqrt{n\left(n-1\right)}} \end{array}$$ (31)

*i.e.*, a linear combination of column i of **P** and a fixed vector p equal to the row sums of **P**. This reveals the decryption key for individual i if p can be guessed.

Thus, in an extreme case, should every individual carry a private variant, or equivalently if n covariates were defined that uniquely identify each individual, then the system can be attacked successfully. While this is an unlikely situation in practice, and one that could easily be avoided, it does suggest that an attack focused on lower-frequency variants might be able to extract useful information. Further, once an individual has been decrypted in this way, close relatives might be more easily identifiable as well.

**Figure 5:**
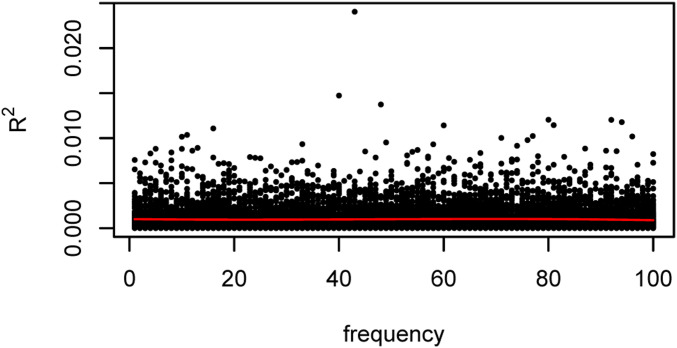
Correlation ${\text{R}}^{2}$ of plaintext and ciphertext dosages as a function of minor allele frequency. Simulations are of genotypes for 1000 subjects with minor allele frequencies in the range (1…100)/1000. Each black dot represents one vector of genotypes. *y*-axis: squared correlation ${\text{R}}^{2}$, *x*-axis: allele frequency. Red line is the smoothed moving average of ${\text{R}}^{2}$.

Equation 30 shows how private variants could be factored out, leaving a smaller orthogonal key representing common variants still to be discovered. However, note that factoring out those individuals with private variants does not reveal useful information about other unrelated individuals, because the remaining columns of P are in the subspace orthogonal to that spanned by the factored columns. In addition, population allele frequencies need not perfectly match those in the sample, so it is not necessarily clear which variants are in fact private. Finally, there is no simple relationship between allele frequency and the correlation between cyphertext and plaintext dosages, as is shown in Figure 5, which plots the squared correlations as a function of allele frequency for simulations.

### Limitations

HEGP leaves the calculation of genetic association unchanged, so should analyze ciphertext in the same execution time as with plaintext. Software that runs off genotype dosage data should run altered since the rotated data are dosage-like. HEGP cannot deal with missing data, which should be imputed first. Another limitation is that it is impossible to analyze subsets of the individuals (*e.g.*, all those of one sex) once they have been encrypted, unless each subset was encrypted separately. However, if a covariate specifying sex is also encrypted then it would be possible to take sex into account when fitting the model.

The simulation of very large orthogonal keys (*e.g.*, for hundreds of thousands of individuals) might also present technical difficulties. A simple solution would be to first permute the rows of D, then group them into a maximum of ∼1,000−10,000 individuals per group, and sample an independent orthogonal key to encrypt each group separately, as described above. The initial permutation would enhance the security of the data by separating potentially similar individuals (permutations are also orthogonal transformations, although in isolation they are useless encryptors as they rearrange phenotype and genotype identically).

For the human depression data, we encrypted the phenotype and genotype dosages in 10 groups of 1000 individuals plus a final block of 664. We computed association across 160 k SNPs using both unencrypted and encrypted dosages. The correlation between the logP values of the tests of association was 0.999. The average absolute difference between the logP values was 0.002. All calculations were performed in R using standard matrix arithmetic. Bearing in mind that usually only the first two decimal places of a logP value are of interest when interpreting the significance of genetic association, we conclude that the numerical inaccuracies introduced by the encryption are negligible.

For the mouse platelet data, the mean absolute difference in logP values for simple association was 6.406e-03, with a maximum of 3.775e-02. We also implemented the mixed model (Equation 13) to confirm that heritability estimates and association *P*-values are numerically stable after encryption. For the mixed model, the mean absolute difference was 3.141e-03 and the maximum 2.635e-02. The mixed-model heritability estimated from the unencrypted data was 0.02534315, compared with 0.025049 after encryption, a discrepancy of 1.1%. We conclude that HEGP does not noticeably affect GWAS results.

### Quantile normalization to improve security

Figure 1D shows that the distribution of ciphertext dosages for a given SNP is almost Gaussian. This suggests that quantile normalizing the ciphertext might improve security. In this scheme, the values in each column of **F** are first ranked and then replaced by their corresponding standard normal quantiles. After quantile normalization the columns of F contain different permutations of the normal quantiles of 1/(n+1)…n/(n+1) that respect the rank orders of the original ciphertext for each column, applying a small nonlinear perturbation to the encrypted genotypes, $F\to F_{q}$. Attacks that exploit nonnormality in the encrypted data would be frustrated, potentially increasing security. A further refinement might iterate an alternating sequence of independent rotations and quantile normalizations.

We evaluated the effects of quantile normalization on the ciphertext mouse genotypes and platelet phenotypes. First, the mean absolute discrepancy for mixed-model association logP values for the plaintext *vs.* HEGP ciphertext was 0.003141 (maximum 0.0263), and the overall correlation of logP values was 0.999: a close agreement. The mean absolute difference between the plaintext and ciphertext dosages (*i.e.*, L1 norm) $[[H-P^{T}F]]$ was $3.561\times 10^{-9}$ (maximum $1.773\times 10^{-6}$). Thus HEGP alone induces only negligible reductions in the accuracy of association statistics and genotypes. However, after encryption and quantile normalization, the mean logP discrepancy rose slightly to $2.402\times 10^{-2}$, maximum $2.257\times 10^{-1}$, but the correlation was still > 0.99. Similarly, the estimated heritability changed 1.3% from 0.02472 to 0.0250. However, the mean absolute error in the decrypted quantile-normalized standardized genotype dosages $[[H-P^{T}F_{q}]]$ rose to 0.03585 (*i.e.*, mean discrepancy 18%), maximum 0.06980.

Our interpretation of this observation is that plaintext dosages correspond to a very special choice of coordinates where the standardized genotype dosages for a SNP are concentrated on three modes depending on the SNP allele frequency. Any random rotation of the genotypes produces coordinates such that the ciphertext dosages closely resemble a Gaussian sample. After rotating into such a coordinate frame it is then possible to make small nonlinear perturbations that have little effect on association statistics or heritability, but degrade the decryption back into the true coordinate system.

We also explored adding further security by quantile normalizing and rounding the encrypted dosages. As would be expected, there is a trade-off between the number of significant digits retained after rounding and the accuracy of association and decryption.

### Logistic regression

So far, we have considered quantitative traits with normally distributed errors, analyzed in a mixed model framework. While case-control studies (*i.e.*, where the phenotype y∈{0,1}) are often analyzed as if they were quantitative traits, under some circumstances it is preferable to use logistic regression, where

$$\mathrm{Pr}\left(y_{i}=1\right)=p_{i}=\frac{e^{{\left(X\alpha +g_{j}\beta_{j}\right)}_{i}}}{1+e^{{\left(X\alpha +g_{j}\beta_{j}\right)}_{i}}}$$ (32)

where ${\left(\mathrm{X\alpha}+g_{j}\beta_{j}\right)}_{i}$ is the ith element of the vector $\mathrm{X\alpha}+g_{j}\beta_{j}$. Write **$X_{j}=\left[X|g_{j}\right]$** and $\alpha_{j}=\left[\alpha |\beta_{j}\right]$. The likelihood for the data at SNP j is

$$\log l=\sum_{i}\log\left(p_{i}^{y_{i}}{\left(1-p_{i}\right)}^{1-y_{i}}\right)+C\left(y\right)=y^{T}X_{j}\alpha_{j}-\sum_{i}\log\left(1+e^{{\left(X_{j}\alpha_{j}\right)}_{i}}\right)+C\left(y\right)={\left(\mathrm{Py}\right)}^{T}\left(PX_{j}\right)\alpha_{j}-\sum_{i}\log\left(1+e^{{\left(X_{j}\alpha_{j}\right)}_{i}}\right)+C\left(y\right)$$ (33)

for any orthogonal matrix P, and where **C(y)** is a function of y only that can be ignored when maximizing the likelihood. Thus, the likelihood function comprises two components, namely $y^{T}X_{j}\alpha_{j}$, which is invariant under orthogonal transformation, and $\sum_{i}\log\left(1+e^{{\left(X_{j}\alpha_{j}\right)}_{i}}\right)$, which is not invariant, instead transforming like $\sum_{i}\log\left(1+e^{{\left(PX_{j}\alpha_{j}\right)}_{i}}\right)$. However, only the first component involves both the dependent and independent variables. This component is shared with the log-likelihood for the normal linear model, which is why fitting a linear model to case-control data generates *P*-values resembling those from logistic regression. It should be clear that case-control data (*i.e.*, $y_{i}\in \left\{0,1\right\}$) are no longer of the same form after an orthogonal transformation, so strictly speaking the likelihood no longer represents a logistic model after transformation. Nonetheless, we can attempt to estimate parameters by maximizing the transformed likelihood (Equation 33).

We fitted the logistic log-likelihood model to simulated SNP data, using untransformed and orthogonally transformed data to assess the change in maximum likelihood parameter estimates under transformation. We found that the estimates changed considerably and therefore orthogonal encryption is not homomorphic for logistic regression, for which we therefore recommend methods such as those of Wang *et al.* (2015).

### The mixed-model linear transformation

Are any nonorthogonal transformations suitable for HE? The mixed-model transformation $A^{-1}$ shares some, but not all, of the invariant properties of the orthogonal group. If we set

$$z_{A}=A^{-1}y,W_{A}=A^{-1}X,F_{A}=A^{-1}F,f_{\mathrm{Aj}}=A^{-1}h_{j}$$ (34)

then the $\text{Var}\left(z_{A}\right)=I$ and the log-likelihood transforms thus:

$$-2\log l={\left(y-\mathrm{X\alpha}-h_{j}\beta_{j}\right)}^{T}V^{-1}\left(y-\mathrm{X\alpha}-h_{j}\beta_{j}\right)+n\text{log}\left|V\right|\to \;{\left(z_{A}-W_{A}\alpha -f_{\mathrm{Aj}}\beta_{j}\right)}^{T}\left(z_{A}-W_{A}\alpha -f_{\mathrm{Aj}}\beta_{j}\right)$$ (35)

Thus, the log-likelihood is preserved so we can extract the mixed-model GWAS *P*-values as before. Moreover, **$A^{-1}$** has $n^{2}$ free parameters, compared to n(n−1)/2 for P, so the decryption problem is presumably harder. Furthermore, it is easily seen that **$A^{-1}$** may be replaced by $PA^{-1}$ for any orthogonal **P** making the decryption harder still. However, there is some loss of information; it is no longer possible to estimate the variance components $\sigma_{g}^{2},\sigma_{e}^{2}$ nor the heritability $h^{2}$. Furthermore, a federated analysis along the lines described above would not give exactly the same *P*-values as would orthogonal transformation followed by a mixed-model transformation applied to the combined data set, because each component study has been transformed separately without guaranteeing that the federated transformed GRM is also the identity; the structure of the federated variance matrix will be of the form

$$V_{C}=\left(\begin{array}{cc} \begin{array}{ccc} I_{1} & ? & ?\\ ? & I_{2} & ?\\ ? & ? & I_{3} \end{array} & \ldots \\ \ldots & \ldots \end{array}\right)$$ (36)

Lastly, linkage disequilibrium between the SNPs is no longer conserved

$$F_{A}^{T}F_{A}=H^{T}{\left(A^{-1}\right)}^{T}A^{-1}H=H^{T}A^{-2}H\neq nL$$ (37)

(because $A^{-1}$ is symmetric).

## Discussion

HEGP has many desirable properties for quantitative genetics. It preserves linkage disequilibrium between genetic variants, and key association statistics including heritability between variants and phenotypes, while obscuring relationships between individuals. However, we do not yet fully understand when HEGP is cryptographically secure. Where private variants are available, decryption is straightforward. While it is simple to remove low-frequency variants and therefore protect against this weakness, the larger question of security remains. We have sketched out several potential attacks but our investigations have not found a workable method. To settle this question, one would need either to find an efficient inversion algorithm, perhaps a version nonconvex minimization under constraints (Bertsimas *et al.* 2010), that recovers the correct genotypes accurately, or alternatively to show there are too many incorrect “genotype-like” decoy solutions far from the true answer, and that therefore the problem is essentially noninvertible. It is likely that the inversion problem might be solvable for small data sets, but much harder for larger ones.

Orthogonal encryption also has the potential weakness that the key space is continuous; in conventional crypto, a small change in the key used leads to a completely different ciphertext. In contrast, a small change to an orthogonal key leads to small changes in the ciphertext. However, at this point, we know of no algorithm that can exploit this. We found that transformed genotypes closely resemble samples from a normal distribution, and so can be replaced by exact normal quantiles with only small effects on accuracy. Hence, we can certainly protect the ciphertext from attacks that rely on non-Gaussianity.

The hardness of the inversion problem depends not only on avoiding private variants, but on choosing a good key. Those sampled from the Stiefel manifold work well at obscuring correlations between plaintext and ciphertext genotypes, such that—as measured by mean correlation across all sites—transformed individuals do not resemble the originals more closely than do simulated individuals with matched allele frequencies. However, it is possible that other measures of genetic similarity between individuals might not be randomized to the same extent. Thus, more work is needed to determine precisely when random orthogonal keys are cryptographically secure. We submit this problem as an open challenge to the community, as described at https://github.com/encryption4genetics.

While HEGP lacks mathematical proof of security compared to normal crypto schemes, most schemes are broken due to weaknesses in implementation (bad random number generators, side-channel attacks, etc.) not algorithms. HEGP has the advantage of an extremely simple algorithm, and is probably immune to side-channel attacks (and to an extent social engineering and rubber-hose cryptanalysis).

Given our current knowledge, we claim that random orthogonal keys provide an encryption scheme where it is, at the least, very difficult to recover individual genetic or phenotypic data. This is at least equal to the level of security of a date shift of medical records, which is also not completely secure but makes it difficult for researchers to identify an individual if they do not intend to do so. Thus, should an effective attack be discovered, orthogonal keys still offer “pretty good genetic privacy” in the sense that they would prevent straightforward copying of information about individuals’ genotypes. We argue that routine orthogonal transformation of genotypes and phenotypes, in combination with existing legal protocols, would enhance security, increase collaboration and data sharing, and thereby accelerate progress.

In summary, we have shown how to make a distinction between public information about genetic architecture and allelic effects, and private information about individuals. This general principle could be applied more widely. We mention two examples. First, to the extent that medical records can be analyzed in a linear modeling framework with a suitable design matrix, orthogonal encryption offers a means to perform federated analyses on orthogonally encrypted medical records. Second, genetic improvement of crops and farm animals could be accelerated. While some germplasm and genetic variation data are in the public domain, commercial breeders are developing new varieties and breeds, and have extensive proprietary genetic and phenotypic data that could be usefully shared using HEGP, so that alleles conferring a beneficial trait could be discovered and published without revealing the genomes of proprietary germplasm under development.

Such a move, toward the idea that an allele’s effects are public property while an individual’s genotypes are private, is more important than the encryption mechanism used to attain it.
